# Systems pharmacology exploration of botanic drug pairs reveals the mechanism for treating different diseases

**DOI:** 10.1038/srep36985

**Published:** 2016-11-14

**Authors:** Wei Zhou, Jinan Wang, Ziyin Wu, Chao Huang, Aiping Lu, Yonghua Wang

**Affiliations:** 1 Key Laboratory of Xinjiang Endemic Phytomedicine Resources, Ministry of Education; Pharmacology Department, School of Pharmacy, Shihezi University, Shihezi, Xinjiang, China; 2 Center of Bioinformatics, Northwest A & F University, Yangling 712100, Shaanxi, China; 3 Institute of Arthritis Research, Shanghai Academy of Chinese Medical Sciences, Guanghua Integrative Medicine Hospital/Shanghai University of T.C.M, Shanghai 200052, China; 4 Institute of Basic Research in Clinical Medicine, China Academy of Chinese Medical Sciences, Beijing 100700, China

## Abstract

Multi-herb therapy has been widely used in Traditional Chinese medicine and tailored to meet the specific needs of each individual. However, the potential molecular or systems mechanisms of them to treat various diseases have not been fully elucidated. To address this question, a systems pharmacology approach, integrating pharmacokinetics, pharmacology and systems biology, is used to comprehensively identify the drug-target and drug-disease networks, exemplified by three representative *Radix Salviae Miltiorrhizae* herb pairs for treating various diseases (coronary heart disease, dysmenorrheal and nephrotic syndrome). First, the compounds evaluation and the multiple targeting technology screen the active ingredients and identify the specific targets for each herb of three pairs. Second, the herb feature mapping reveals the differences in chemistry and pharmacological synergy between pairs. Third, the constructed compound-target-disease network explains the mechanisms of treatment for various diseases from a systematic level. Finally, experimental verification is taken to confirm our strategy. Our work provides an integrated strategy for revealing the mechanism of synergistic herb pairs, and also a rational way for developing novel drug combinations for treatments of complex diseases.

Traditional Chinese medicine (TCM) has been widely used for treating diseases over thousands of years and recognized as a valuable and readily available resource to conventional medicine. Many inspiring experiences have demonstrated that combining diverse medicinal herbs could be much more efficient with better curative effects and fewer side effects^1^. Thus multi-herb therapy has been considered as an essential component of traditional medicine systems in China and many other countries.

In TCM, multi-herb prescriptions often include special herb pairs that are claimed to be assembled and interpreted unique combinations of the traditionally defined herbal properties, involving mutual enhancement, assistance and restraint^2^. The therapeutic effects of herb pairs are usually attributed to the synergistic effects achieved by using a pair of herbs with ingredients of similar therapeutic actions^3^. For example, *Radix Salviae Miltiorrhizae (S. miltiorrhizae*, Danshen in Chinese), one of the main components in many herb pairs, is often in combination with other herbs for promoting blood circulation, relieving blood stasis, clearing heat from the blood, resolving swelling and tranquillizing the mind. More interestingly, *S. miltiorrhizae* shows strong synergisms with many other herbs in clinical trials for treatment of various diseases, such as the Danshen-Yimucao (*HerbaLeonuri*, *H. leonuri)* pair, which has been widely used for coronary heart disease (CHD) treatment^4^. The cooperation of Danshen and Xiangfu (*Cyperus Rotundus*, *C. rotundus*) has an effect on treating dysmenorrhea, and the herb pair of Danshen-Zelan (*Eupatorium Japonicum Thunb*, *E. Japonicum*) is recorded to treat nephrotic syndrome^4^. However, although these herb pairs in ethnopharmacology have been widely explored and their effectivenesses are gradually proved in modern times, the exact molecular synergism underlying such multi-component synergy still remains unclear.

Based on research and experience in the context of multicomponent therapeutics, some experiments in a case-by-case method are usually proposed^5^. For instance, a novel antimalarial drug fosmidomycin has been shown to act synergistically with clindamycin against Plasmodium falciparum both *in vitro* and *in vivo*^6^. Subsequently, High-Throughput screening (HTS) has emerged as a powerful tool to identify potential therapeutic targets, therapeutic lead compounds and possible drug combinations based on the classical and chemical genetic approaches^7^. It is reported that a combination high throughput screening (cHTS) platform has been developed to systematically and efficiently investigate combination drugs with synergistic activity^8^. However, due to the diversity of chemical components and the complexity of multicomponent synergistic mechanisms of action, it is extremely difficult to screen all possible drug combinations for all possible indications. In addition, these “blind” approaches including molecular biology not only are expensive and time consuming, but also limit the full investigation of mechanisms of drug combinations from a system perspective.

Alternatively, the emergence of computational methods provided a chance to investigate the complex mechanisms of drug action which are capable of overcoming the challenges brought about by experiment. Recently, several network-based approaches have been developed and applied to explore the potential synergy mechanism for drug combinations. For example, Chen *et al.* established a Network-based Laplacian regularized Least Square Synergistic drug combination prediction method (NLLSS) to quantitatively identify potential synergistic drug combination based on the information of known synergistic drug combinations, unlabeled drug combinations, drug-target interactions and drug chemical structures^9^. Sun *et al.* provided implications for promoting combination therapy of cancer by employing module-based kinetic modeling approach which was devoted to investigating the signaling crosstalk-mediated mechanisms of drug resistance, as well as the relative efficacy and synergism of drug combinations^10^. Li *et al.* proposed “network target”-based method which aimed at using network analysis to establish an algorithm termed NIMS (Network target-based Identification of Multicomponent Synergy) for investigating the potential mechanisms of synergistic agent combinations from a network target perspective^11^. Wu *et al.* made full use of network-based systems biology approach to identify effective drug combinations based on high throughput data^12^. In addition. Wang *et al.* developed a network pharmacology method to uncover the pharmacological synergy in herbal combinations which is in favour of comprehensive understanding the mechanisms of combinatorial therapy and prediction of new drug combinations^13^. Yao *et al.* used Ma-huang Decoction as a probe to systematically decipher the combination principles (“Jun-Chen-Zuo-Shi” rule) of Traditional Chinese Medicine at the point of systemic view^14^.

The ultimate ideal of network-based approaches is to identify and analyze the drug-target and drug-disease interactions for investigating whether a drug could interact with a target in a disease at the molecular level. In this case, several computational methods are becoming more and more powerful to predict and analysis such complex relationships, which have always been the major objective of bioinformatics technology^13^,^15^,^16^,^17^,^18^. For instance, a method of NRWRH (the method of Network-based Random Walk with Restart on the Heterogeneous network) has been developed which takes full advantage of the tool of the network for data integration to predict potential drug-target associations on a large scale^19^. Yang *et al.* proposed a computational method to predict multiple target optimal intervention (MTOI) solution based on systematically analyzing the best transformation of a disease network from the disease state into desired state^20^. Campillos *et al.* developed a computational algorithm for finding drug-target associations based on the information of drug side-effect similarity measure^21^. A computational method PREDICT using multiple drug-drug and disease-disease similarity measures to directly predict novel drug-disease associations for both FDA approved drugs and experimental molecules on a large scale was proposed by Gottlieb *et al.*^22^. Furthermore, in our previous work, we have developed a robust multiple drug-target interactions prediction (DTpre) model to identify the potential targets for a given molecule based on support vector mechanic (SVM) and random forest methods (RF)^23^.

Although these computational models have shown their significant improvements in identifying new therapeutic targets of existing drugs and exploring potential synergistic drug combinations compared with the experimental methods. However, they still have some limiting factors due to the complexity of multicomponent interactions and pharmacological synergistic mechanisms of action in herbal medicines. Firstly, some of these methods often only work on small drug sets limited by the high-cost. Generally speaking, drugs are combined based on their properties which mainly include their targets and pharmacological features^21^. Therefore, the incompleteness of molecular networks and the insufficient of drug properties limit the reliability and accuracy of such methods to investigate the complicated mechanisms of TCM. Secondly, some approaches always constructed the drug-target interaction model as a simple binary classification problem which do not reflect the real-life case in drug-target interactions, since complex diseases are multifactorial in nature that tend to be associated with multiple drugs and target proteins. Finally, these methods only focus on the prediction of drug-target or drug-disease interactions individually, they ignored the concept that TCM emerged and were considered as a holistic view of the human being which included the integration and dissection of pharmacokinetics profiles, pharmacological features and underlying synergistic mechanisms of action associated with drugs, as well as the investigation of various complex relationships, such as drug-target interactions, drug-disease connections, target-disease associations, drug-pathway connections and from molecular to system level. In comparison with these approaches, systems pharmacology is likely to be a comprehensive method in providing a wealth of information for integrated multi-scale analysis of synergistic mechanisms of multi-component drugs and interrelationships of complex networks by using both computational and experiments techniques^24^,^25^,^26^. With this understanding, the application of systems pharmacology in herb pairs may effectively and systematically help to elucidate the synergistic mechanism of drug combinations, including the build-up of pharmacological networks, so as to promote the discovery of new therapeutic indication of existing drugs and potential synergistic drug combinations.

Therefore, in this study, three representative *S. miltiorrhizae*-dominated synergistic drug pairs (Danshen-Xiangfu, Danshen-Yimucao, Danshen-Zelan) were collected. We provide an integrated strategy by combining active ingredients screening, pharmacology feature mapping, multiple targeting, network pharmacology techniques and experimental verification to systematically revealing the herb synergism for drug pairs^13^,^15^,^16^,^17^. The systems analysis of different *S. miltiorrhizae* pairs would also provide a novel and efficient way to further explore why different *S. miltiorrhizae* combinations have contributed to controlling various diseases. Knowledge of the molecular mechanism of the synergistic combinations of herb pairs based on systems pharmacology not only facilitates the development of novel drug combinations that are individually subtherapeutic but efficacious in combination, but also opens up new ideas to fundamentally elucidate the scientific connotation of multiple systems of TCM, so as to better explore the complex therapeutic mechanism at the systems level.

## Materials and Methods

### Design

**Figure 1:**
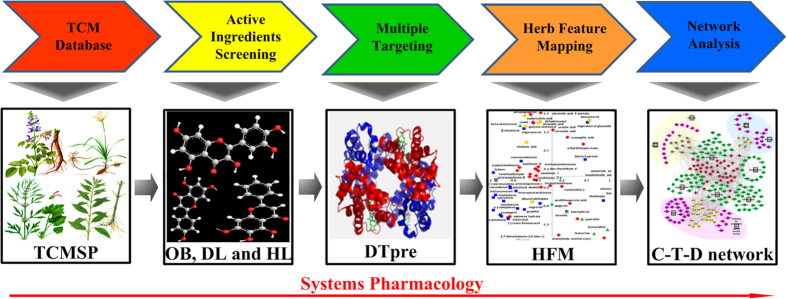
Workflow for systems pharmacology-based botanic drug pairs study. (1) obtainment of chemicals for herbs from TCMSP database; (2) screening the potential active compounds with OB, DL and HL; (3) target identification and validation; (4) investigation the chemical and pharmacological features of the herbs; (5) building and analysis of drug-target-disease network.

To address the challenges in the study of the molecular synergetic mechanism and combinatorial principle of different herbal pair to treat different types of diseases, we developed an integrated strategy based on a systems pharmacology framework which is shown in Fig. 1. In addition, the computational efficiency of algorithms used in our strategy are listed in Table S1.

### Herb data source

Previously, we built a TCMSP: Traditional Chinese Medicine Systems Pharmacology Database and Analysis Platform (http://lsp.nwsuaf.edu.cn/tcmsp.php), which collected a total of 12144 chemical ingredients for all the 499 herbs registered in the Pharmacopoeia of the People’s Republic of China^27^. For Danshen-Yimucao, Danshen-Xiangfu and Danshe-Zelan, we have selected all available ingredients for each of four herbs, 204 for *S. miltiorrhizae*, 52 for *H. leonuri*, 104 for *C. rotundus* and 16 for *E. Japonicum*. We also conducted *in silico* analysis to identify and determine the major molecular descriptors which were calculated using DRAGON 5.4 program (http://www.talete.mi.it/index.htm). The detail information is provided in the TCMSP database, which is freely available online.

### Active ingredients screening

High oral bioavailability is often a key indicator for a molecule to be therapeutic agent. Recently, we have developed an in-home system OBioavail1.1 with high accuracy^28^. In this work, the compounds with OB ≥ 50% were selected as the candidate compounds since average bioavailability for oral administration is 30% with 10–50% variability according to clinical studies. The threshold determination is based upon two careful considerations. Firstly, get as much information as possible from the four herbs by using the least number of chemicals. Secondly, the obtained model (OBioavail1.1) can be rationally interpreted by the reported pharmacological data.

In addition, discriminating “drug-like” molecules from enormous number of molecules is also one of the prime focuses of the current research. For the purpose of filtering out the drug-like molecules, we have developed a database-dependent model to discriminate between drug-like and non-drug-like chemicals using the Tanimoto coefficient^29^. Using 0.5 as the threshold, the compounds with drug-likeness (DL ≥ 0.5) would be picked out for further analysis. The threshold of DL is determined upon the fact that the mean value of DL for all 6511 molecules in DrugBank is 0.18^25^.

Furthermore, half-life (t_1/2_) of a drug is the time required to reduce the drug concentration by half, which is arguably the most important property as it dictates for the timescale over which the compound may elicit therapeutic^30^. A new *in silico* model has been developed to predict half-life (preDHL) of drugs with good overall accuracy of internal validation (85.21%) and external validation (86.05%) by using the C-partial least square (C-PLS) algorithm^31^. In our present paper, the compounds with the half-life value ≥4 hours were selected by the preDHL model since a medication generally has a 4 hour half-life to reach a consistent level in the body.

### Multiple targeting strategy

#### Drug-target interaction identification

Comprehensively determining compound-target interaction profiles and mapping these on signaling and metabolic pathways will become increasingly necessary for elucidating the mechanism of drug action. *In silico* prediction of target profiles of small molecular compounds especially is a critical step for the study of TCM system pharmacology.

We proposed a systematic approach which effectively integrated data mining, chemogenomic, pharmacological and statistical methods to identify molecular targets of active compounds. First of all, a text mining for all target proteins was carried out in HIT (Herbal Ingredients’ Targets database, http://lifecenter.sgst.cn/hit/)^32^, TTD (http://bidd.nus.edu.sg/group/ttd/)^33^ and DrugBank^34^ to obtain a more complete and greater accuracy view on the drug-target interactions. All compound-target interactions from these databases were known and supported by published literatures. Secondly, the virtual chemical Engerprint Similarity Ensemble Approach (SEA, http://sea.bkslab.org/)^35^, information integration method STITCH (http://stitch.embl.de/)^36^ and omics-based Ligand-Target Chemogenomic model (LTC) were applied to predict the biological targets of active ingredients^23^. Recently, we have developed a robust DTpre model to identify the potential drug-target interactions^23^. DTpre model is a chemogenomic integrated method that effectively combines the chemical, genomic and pharmacological information based on SVM and RF, and this optimal model exhibited good performance of predicting the compound-target interactions, with concordance of 82.83%, sensitivity of 81.33%, and specificity of 93.62%, respectively^18^. Generally speaking, setting the score to at least 0.5 exhibits a good affinity prediction of the compounds to a target protein^37^. Therefore, in this study, the targets with the value of SVM score ≥ 0.8 and RF score ≥ 0.7 were selected as the final predicted targets for improving the accuracy of prediction. Finally, the systematically evaluated target proteins were further subjected to TTD, PharmGkb (http://www.pharmgkb.org)^38^, UniProt database (http://www.uniprot.org/)^39^ and Comparative Toxicogenomics Database (CTD, http://ctdbase.org/)^40^ to obtain a more complete and greater accuracy view on the drug-target interactions. These predicted target profiles will be collated into an integrative target profiles for in-depth validation.

To investigate the relationships between herbal targets and diseases, we sent the target information of herb to TTD database to mine target-related diseases. Then map herb and all known targets to PharmGKB, TTD and CTD databases to build the connections with diseases. Finally, all the information was sent to Medical Subject Headings (http://www.nlm.nih. gov) for further identification of disease categories.

### Drug-target interaction validation

#### Molecular docking

Molecular docking is a computational tool frequently used to predict the predominant binding mode(s) of a small molecule compound candidate with a protein target of known three-dimensional structure. This approach has been found to be fairly successful in searching high-dimensional spaces effectively and uses a scoring function that correctly ranks candidate dockings. Therefore, considering the above obtained results, it was worthy to perform molecular docking simulation, hence further validating the compound-target binding. The Lamarckian genetic algorithm^41^, inculcated in the docking program AutoDock software^42^, as one of the most widely used docking programs in computational binding studies was employed to satisfy the purpose. All the crystal structures of candidate targets were directly retrieved from the RCSB Protein Data Bank (www.pdb.org)^43^ with their resolutions being carefully examined. In addition, the proteins without crystal structures were performed by the Swiss-Model Automated Protein Modeling Server with default settings (http://swissmodel.expasy.org/). Prior to the docking studies, polar hydrogen atoms, Kollman charges and atomic solvation parameters were added to the protein, the Gasteiger charges were assigned with the nonpolar hydrogens merged^44^. The docking calculations were carried out by using 60 Å × 60 Å × 60 Å 3D grids centered around the ligands binding site with a 0.375 Å grid space. In our previous studies, the potential protein targets of TCM were successfully predicted with a good compound-protein binding affinity based on the threshold level of ≤−5.0 kcal/mol^25^,^29^,^45^. Therefore, in the present work, target docking scores ≤ −5.0 kcal/mol as an empirical threshold were also selected as the potential targets for further analysis.

#### Molecular dynamics simulation

To further validate the drug-target interactions, molecular dynamic (MD) simulations were performed with the Amber 10 suite of programs using the standard AMBER99SB force field^46^,^47^. The partial atom charges and parameters of the ligand are derived from the AM1-BCC procedure^48^ and the generalized Amber force field (GAFF)^49^. The prepared systems were neutralized by adding sufficient Na^+^/Cl^−^ counterions and the truncated octahedron box of TIP3P water molecules was added to a distance of at least 10 Å buffer from any solute atom using the tLeap module of Amber 10. The cut-off distance of 8 Å was used to compute the non-bonded interactions and the Particle-mesh-Ewald method (PME) was employed to treat the long range electrostatic interactions^50^. Standard techniques for periodic boundary conditions were also used and all bonds containing hydrogen atoms were constrained using the SHAKE algorithm.

After the processes above, the obtained standard equilibration protocol was then subjected to 500 steps of steepest descent energy minimization followed by 1000 steps of conjugate gradient algorithms for monitoring the convergence and structure analysis. Each of these structures was gradually heated from 0 to 300 K over a period of 50 ps and the force of 2.0 kcal/mol/Å^−2^ on the heavy atoms of the complex was restrained during density equilibration. The whole system equilibration was performed a 50 ps pressure-constant period to raise the density and a 500 ps MD simulation at constant pressure was then conducted. Production phase of 5 ns was carried out using MD simulation at 300 K and one atmospheric pressure for each system, respectively. The step size of 2fs was used for the simulations and the coordinates of the trajectories were saved every 2 ps.

#### Binding free energy calculation

The energy of the protein-ligand binding was calculated by the Molecular Mechanics-Poisson Boltzmann Surface Area (MM-PBSA), SANDER and NMODE modules integrated into Amber^51^. The binding free energy (Δ*G*_*bind*_) was approximated by the following equations:


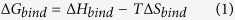



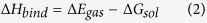


T represents the temperature of the system at 300 K, while Δ*H*_*bind*_ is defined as the average enthalpic contribution. *T*Δ*S*_*bind*_ is the contribution of entropy changes to the binding free energy, which was generally computed by normal-mode analysis based on the NMODE module in Amber. The enthalpy term in above equations is dissected into sub-energy terms:


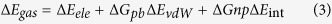



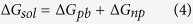



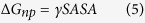


where Δ*E*_*gas*_ is the potential molecular mechanical gas-phase energy which is determined as the sum of electrostatic (Δ*E*_*ele*_), van der Waals (Δ*E*_*vdW*_) and internal energies (Δ*E*_int_) by using the SANDER module of Amber 10. Δ*G*_*sol*_ is the solvation free energy for transferring the solute from vacuum into solvent, which is computed using the PB/SA model and is a sum of electrostatic (Δ*G*_*gb*_) and non-electrostatic (hydrophobic) contributions (Δ*G*_*np*_). Δ*G*_*pb*_ was computed using the PBSA module of Amber 10 with the default cavity radii set. The Δ*G*_*np*_ was obtained using eq. (5), in which *SASA* is the solvent-accessible surface area (Å^2^) calculated by the linear combination of pairwise overlaps model^52^. In our calculations, the value for surface tension(γ) was set to 0.0072 kcal.mol^−1^ Å^−2^. Generally, the low binding free energy is indicative of high binding affinity of the compounds to their targets^45^ Thus, as a conservative starting point, the Δ*G*_*bind*_ of compounds and their corresponding targets was set under the threshold of −10.0 kcal/mol, an empirical threshold value referred to previous studies^45^,^53^.

#### Herb feature mapping

To investigate the differences in chemistry and pharmacology among the three herbs pairs, we developed a method to visualize all ingredients in the four herbs according to their chemical features, termed herb feature mapping (HFM) method. By considering eight representative drug-related physicochemical properties including molecular weight (MW), number of rings per molecule (nCIC), octanol-water partition coefficient (MlogP), hydrogen bond donors/acceptors (nHDon and nHAcc), number of rotatable bonds (RBN), hydrophilic factor (Hy), and topological polar surface area (TPSA)^54^. The distribution of the active components from these herbs in chemical space can be visualized by principal components analysis (PCA), which projects the high-dimensional data into a low-dimensional space, allowing for clear imaging of the variations between different herbal ingredients. In addition, PCA makes it possible to identify the most important directions of variability in a multivariate data matrix and to present the results in a graphical plot. More importantly, we can map the pharmacological activities of each ingredient in four herbs to determine whether herbal ingredients can be distinguished according to their chemical feature.

#### Network construction and analysis

To better elucidate the holistic pharmacology functions and therapeutic effects of three *S. miltiorrhizae*-dominated herb pairs, compound-target-disease network (C-T-D network) analysis was performed. The C-T-D network was constructed by linking the active compounds, candidate targets and their related disease of three herb pairs, which aimed to develop a systems-comparison of different *S. miltiorrhizae* pairs control various diseases so as to further interpret the mechanism of drug action and comprehensive relationships between the diseases and botanic drugs. In addition, in order to understand the possible biological functions of each herb, the functional analysis of these C-T-D networks was further examined. Finally, the combinational rationales of the three *S. miltiorrhizae* herb pairs were explained according to the detailed analysis of enriched biological functions. All the networks were generated by Cytoscape 2.8.1, which is an open source software project for integrating biomolecular interaction networks with high-throughput expression data and other molecular states into a unified conceptual framework (http://www.cytoscape.org/)^55^.

#### Experimental verification

To verify the reliability of our method, an *in vitro* assay was conducted to further investigate the compound-target binding interactions following the manufacturer’s instructions. These compound-target interactions were randomly selected as the seed agent which readily available on the market. Compounds salvianolic acid A, kaempferol, quercitin and luteolin were purchased from Yitai Technology Ltd. (Wuhan, China). The standardized compound which has effect on F10 (Factor Xa) was measured in the use of SensoLyte^®^ Rh110 Factor Xa Assay Kit (AnaSpec, CA, USA) following manufacturer’s instruction. The inhibitory effects of PTGS2/COX2 (Prostaglandin G/H synthase 2) were tested using Colorimetric COX (ovine) inhibitor screening assay kit (Cayman Chemical, Ann Arbor, MI, USA). In addition, targets PIK3CG (PI3-kinase p110-gamma subunit) and MAOA (Monoamine oxidase A) were respectively purchased from Merck Millipore Corporation, Germany and BioVision, USA. The purity of all compounds is >98%. All the compounds dissolved in ethanol (10%) were freshly stored to avoid loss of activity if long placed. The 50% inhibition concentration (IC_50_) values were calculated from survival curves using the Bliss method with three independent determinations.

#### Statistical analysis

The one-way analysis of variance (ANOVA) and Student’s t-test were applied to investigate and compare means of the parameters being inhibitory influenced. Student’s t-test was used to demonstrate the means of two groups in comparison. ANOVA test was performed to compare the means of multiple groups. Differences were considered statistically significant when values of p-value less than 0.05. All values were reported as the mean ± standard error of the mean (S.E.M.) of three samples in parallel.

## Results and Discussion

### Combinatorial screening for each herb

In most cases, TCMs are orally administered. It is believed that most compounds in the mixture fail to reach to the cellular targets as they lack appropriate pharmaceutical properties, such as favorable oral bioavailability^28^ and drug half-life^30^. Therefore, a combinatorial screening for drug-like properties should be indispensable to determine whether a compound is pharmaceutically active in a complex TCM mixture. Our analysis for the herb pairs shows that 92 compounds which account for 24.5% of all the 376 chemicals have satisfactory properties with the filter criterion: overcoming 66.7% (2/3) of the oral bioavailability (OB  ≥ 50%), favorable drug-likeness property (DL ≥ 0.5) and half-life (HL ≥ 4). The detailed information is provided as follows (Table S2).

#### Radix Salviae Miltiorrhizae

*S. miltiorrhizae* (belongs to *Labiatae*), the dry root of *Salvia miltiorrhizaBge*., is a widely used herb for promoting circulation and improving blood stasis, resolving swelling and tranquilizing the mind. It is frequently used in decoction preparations either individually or in combination with other TCM for the treatment of cardiovascular diseases in clinic, including CHD, hypertension, diabetes, atherosclerosis and chronic heart failure, in respect of efficacy and fewer side effects^56^.

In this herb, total 46 (22.5% of all 204) bioactive components from *S. miltiorrhizae* meet our filter criteria (Table S2), including tanshinone IIb (OB = 65.26%, DL = 0.45 and HL = 23.48), cryptotanshinone (OB = 52.34%, DL = 0.40 and HL = 17.30), salvianic acid A (DL = 0.70, HL = 5.21), salvianolic acid C (DL = 0.81, HL = 13.62), salvianolic acid J (DL = 0.72, HL = 5.77), tanshinol I (OB = 56.97%, DL = 0.52 and HL = 5.15) and so on. Searching the molecular mechanism in depth, most of them have been reported as bioactive ingredients and well demonstrated related to CHD. For example, tanshinone IIb, a primary active constituent from *S. miltiorrhizae*, widely used in the treatment of stroke and coronary heart disease in Asian countries^57^. Cryptotanshinone can protect the myocardium against ischemia-induced derangements by eliciting a significant enhanced recovery of the contractile force upon reoxygenation^58^. In addition, salvianolic acids from *S. miltiorrhizae* can increase cerebral blood flow after ischemia and inhibit thrombosis, thromboxane B2 formation and platelet aggregation^59^. Salvianic acid A (DL = 0.70, HL = 5.21) as one of the most effective components of *S. miltiorrhizae* has been widely used in treating CHD^60^. Notably, although salvianolic acid B has low OB and DL (OB = 3.01%, DL = 0.41 and HL = 18.53), it is one of the most abundant constituents in Salvia species, which may show good pharmacological effects on atherosclerosis^61^ and platelet aggregation^62^ due to not only the salvianolic acid B itself but also its metabolites. The evidence has been proved that this compound is water soluble and can be rapidly metabolized *in vivo* to salvianic acid A and quickly excreted into bile after oral administration^63^.

#### HerbaLeonuri

*H. leonuri* (Yimucao in Chinese), also known as the “mother-benefiting herb”, is the aerial part of *Leonurus japonicas Houtt. (L. artemisia*, *L. heterophyllus*, Lamiaceae)^64^. *H. leonuri* mainly acts on the liver, pericardium and urinary bladder channels to promote blood circulation by removing blood stasis and qi stagnation when entering the blood system^65^. This herb has the effects of promoting tissue regeneration, increasing coronary flow and microcirculation, enhancing urine excretion and reducing swelling, decreasing heart rate and blood hyperviscosity, as well as antioxidant. Therefore it is used to treat such diseases as cardiovascular diseases, postpartum blood stasis and ref. 64 to name only a few.

The major ingredients in *H. leonuri* include alkaloids, flavonoids, diterpenoids, saponin, organic acids and so on^66^. In this herb, about twenty-one percent (11 of 52) of the compounds in *H. leonuri* are obtained, which are shown in Table S2. Among them, the alkaloids in *H. leonuri*, predominantly leonuridine (OB = 106.15%, HL = 6.42) and leonurine (OB = 19.12%, DL = 0.20, HL = 4.71), were proved to be biologically active components *in vivo* and *in vitro* pharmacological tests^67^. In particular, although leonurine has low OB = 19.12% and DL =  0.20, it exhibit significant biological activities^68^. It has recently been confirmed to be beneficial against cardiovascular diseases, including acute and chronic post-myocardial infarction, ischemic stroke^69^. Except for the alkaloids, the apigenin (OB = 69.81%, HL = 16.62), kaempferol (OB = 69.31%, HL = 12.68), quercitin (DL = 0.77, HL = 14.40) and rutin (DL = 0.68, HL = 16.13), which belong to the flavonoids, have been shown a wide spectrum of biological activities including anti-inlammatory, anticancer, and antioxidative effects^70^,^71^.

#### Cyperus Rotundus

*C. rotundus* (Xiangfu in Chinese), the root of *Cyperus rotundus Linn*, is widely used for the treatment of spasms, bowel and stomach disorders, dysmenorrhea, and inflammatory diseases in India, China, Japan, and Korea^72^. When we screen the active compounds of *C. rotundus*, 28compounds accounting for 26.9% of the total 104 compounds meet our certain criteria as shown in Table S2, such as rotundenol (OB = 74.95%, DL = 0.84 and HL = 7.51) and khelloside (OB = 74.96%, DL = 0.72 and HL = 14.34). Interestingly, among these 28compounds, flavonoids such as luteolin (DL = 0.78 and HL = 15.94) have reported anti-oxidant, anti-inflammatory and anti-allergic activities^73^. β-sitosterol (DL = 0.75 and HL = 5.37) was found to possess potent anti-inflammatory activity against both the tests, similar to hydrocortisone and oxyphenbutazone when administered intraperitoneally^74^. Nootkatone (DL = 0.75 and HL = 4.39) was found to have the most potent inhibitory effect on platelet aggregation^75^. At last, alpha-cyperone was also included since this compound exhibits significant therapeutical effect on dysmenorrheal and plays an important role in the inflammation process^76^, although their OB, DL might not be acceptable (OB = 35.37%, DL = 0.10).

#### Eupatorium Japonicum Thunb

The extract of *E. japonicum* (Zelan in Chinese, Labiatae) has been used for centuries as an oriental traditional medicine. This crude herb is a rich source of flavonoids, coumarins, terpenoids and tannins and is used for the management of menstrual disorder and inflammatory disease^77^. It has been known to have anti-oxidative and anti-inflammation effects^78^. Only 7 compounds are obtained from *E. japonicum* (Table S2), which include oleanolic acid (DL = 0.76 and HL = 4.83) and ursolic acid (DL = 0.76 and HL = 4.86), the major ingredients in this herbal. Oleanolic acid and ursolic acid have been long-recognized to have anti-inflammatory and anti-hyperlipidemic properties in laboratory animals^78^. They are also relatively non-toxic, and have been used in cosmetics and health products^79^. In the remaining compounds, daucosterol and β-sitosterol are also the main anti-inflammatory effective constituents^74^ with high DL and HL (daucosterol: DL = 0.63 and HL = 6.88, β-sitosterol: DL = 0.75 and HL = 5.36). It has also been reportedcholanic acid (DL = 0.59 nd HL = 5.27) has a strong antibacterial property^80^ and dibutyl phthalate (OB = 64.54% and DL = 5.41) could affect reproductive function^81^.

### Target identification and validation

Generally, the way for a drug to display its pharmacological effects is via its interactions with one or more protein targets. In this section, based on our previous developed robust DTpre model and TCMSP database^27^,^23^, the binding affinity of three Danshen pairs to their targets of interest incoronary heart disease, dysmenorrheal and nephrotic syndrome was investigated. Except for seven compounds (d-borneol, cedrol, shanzhiside methyl ester, uvaol, (−)-trans-Pinocarveol, dimethyl tetrasulfide, and dimethyllithospermate) that have no targets, total 85 candidate ingredients yielded 150 protein targets and 827 ingredient-target interactions were predicted for the Danshen, Yimucao, Xiangfu and Zelan (Table S3).

To verify the reliability of the candidate targets, molecular docking was further performed to calculate the compound-target binding interactions, and only those with binding free energy ≤ −5.0 kcal/mol were selected as the potential targets. This leads to the number of interactions between compounds and targets is sharply reduced from 827 to 562. As a result, only 67 targets were collected and retained (Table S3), which linked with 70 ingredients (Table S4). Among these proteins, 43, 31, 32 and 25 were recognized as the targes of Danshen, Yimucao, Xiangfu and Zelan, respectively. The detail information for the compound-target interactions are shown in Table S3.

In order to estimate whether the results obtained by the molecular docking analysis were robust or fortuitous and reproduce the actual behavior of real molecules in motion, MD simulation and binding free energy methods are carried out to reproduce the actual behavior of the binding of compound and targetin motion. Therefore, in our study, six systems include CCNA2-YMC11, ESR1-DS3, MAPK14-ZL4, NCOA2-ZL4, PGR-XF7 and RXRA-DS38 were collected to perform the analysis although DTpre model and molecular docking might exhibit more reliable results. The selected proteins of ten systems are all important targets and their corresponding compounds are all critical ingredients of the three *S. miltiorrhizae*-dominated herb pairs with less than −5.0 kcal/moldocking binding energy as well.

The MD trajectories of ten compound-target complexes in a solvated system were successfully run for 5 ns scale. The stability and equilibration of MD simulations were examined by the root mean square deviation (RMSD) of protein Cα backbone atoms and the initial structures were monitored through the phase of the simulation. The MM-PBSA method had been used to calculate the absolute binding free energies of the ten systems based on the single trajectory protocol. The calculated energy contributions to the binding free energies and components are listed in Table 1. It is shown that the binding free energies of ten systems are low (−17.58~−33.17 kcal/mol), which implies the high binding affinity of the three compounds to their targets, as models with a low value of free energy are generally considered to be more stable than those with high values. As is shown in Table 1, both the intermolecular van der Waals and the electrostatic interaction energies have shown significant contributions in the binding. The overall electrostatic interactions energies, (Δ*E*_*ele*_ + Δ*G*_*pb*_) are positive and unfavorable while the van der Waals and hydrophobic interaction contributions (Δ*E*_*vdW*_ + Δ*G*_*np*_) are in favor (negative) of the binding. These results are consistent with the fact that when a ligand transfers from the solvent to the binding pocket, the electrostatic contributions are unfavorable to ligand binding^82^, which demonstrates that the current analyses by MD simulations are reliable.

**Figure 2:**
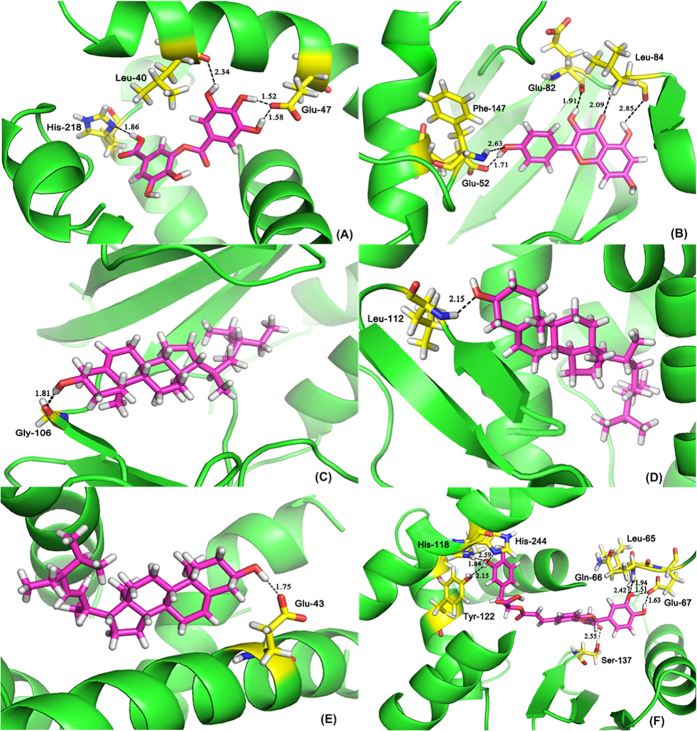
Molecular models of YMC11, DS3, ZL4, XF7 and DS38 in the binding sites of CCNA2, ESR1, MAPK14, NCOA2, PGR and RXRA. Model compounds and residues within 2.85 Å are shown as stick representation. Hydrogen bonding interactions are shown as black dashed lines. Yellow and magenta: carbon; red: oxygen; blue: nitrogen; cyan: hydrogen. (**A**) Representative interactions between YMC11 and CCNA2. (**B**) Representative interactions between DS3 and ESR1. (**C**) Representative interactions between ZL4 and MAPK14. (**D**) Representative interactions between ZL4 and NCOA2. (**E**) Representative interactions between XF7 and PGR. (**F**) Representative interactions between DS38 and RXRA.

The binding mode of six systems is taken as the examples for analysis in this work, which demonstrated that all active compounds were well accommodated inside the binding pocket of their corresponding target proteins. Both functional groups played important roles in the binding activity and are involved in the compound-target interactions with key residues, as shown in the Fig. 2. For example, in Fig. 2A, the key residues that form the binding pocket of CCNA2 are Glu 52, Phe 147, Glu 82 and Leu 84, and the model compound is anchored in the binding pocket via several H-bonds. The distance between hydrogen of YMC11 and the oxygen atom of Glu 52 is 1.71 Å, while the oxygen atom of the hydroxyl group is involved in hydrogen bonding with the backbone of Phe147 (2.63 Å). The –OH group of small molecule is H-bonded to the backbone carbonyl of amino acid Glu 82 at a distance of 1.91 Å, which results in appropriate binding mode. In addition, two hydrogen bonds are formed between the oxygen atom of CCNA2 and the –NH group in Lue84 with a distance of 2.09 Å, and between the hydroxyl group of CCNA2 and the carbonyl group of Lue84 (2.85 Å), respectively.

Figure 2B shows the conformation derived for DS3 with the binding site of ESR1, in which four hydrogen bonds are produced. The two hydrogen atoms of DS3 form two H-bonds with the side chain nitrogen of His 218 (–OH···N, 1.86 Å) and the oxygen atom of backbone carbonyl group in Leu 40 (–OH···O, 2.34 Å), respectively. Another two hydrogen bonds are formed between the hydroxyl group H atoms of DS3 and oxygen atom of Glu 47 (–OH···O, 1.52 Å, –OH···O, 1.58 Å), the hydroxyl group in the ligand serves as an H-bond donor and amino acids as H-bond acceptor. The best possible interacting mode of compound ZL4 with MAPK14 is described in Fig. 2C. The analysis shows that Gly 106 is the important residue present at the active site. The only H-bond (1.81 Å) is built between hydroxyl group H atom of ZL4 and the oxygen substituent of Gly 106. The interaction significantly enhances the stability of the compound-target complex with the high score of 7.57.

The binding mode of compound ZL4 is shown in Fig. 2D, involving a crucial residue Leu 112 in the active site. It is shown that the small molecule ZL4 is well located in the center of the NCOA2 active site, and the docked model of the complex forms hydrogen bond with –NH group of Leu 112 at a distance of 2.15 Å. For the system XF7- PGR (Fig. 2E), Glu 43 is identified as the key residue, located within the binding pocket of PGR. A proper H-bond (1.75 Å) is built between the hydrogen atom of hydroxyl group and O-atom of Glu 43, while the hydroxyl of the XF7 serves as an H-bond donor and Glu 43 as H-bond acceptor. The Fig. 2F clearly reflects that good interactions are found between DS38 and binding residues of RXRA. The key residues that form the binding pocket of RXRA are Tyr 122, His 118, His 244, Gln 66, Glu 67 and Ser 137, anchored in the compound via several H-bonds. In active site, the DS38 is capable of making two H-bonds with the hydroxyl group of Tyr 122 (–OH···O, 2.15 Å) and nitrogen atom of His 244(–OH···N, 2.59 Å) as H-bond donors, while the oxygen of the identical hydroxyl group in the compound acts(delete) form one H-bond with the -NH of His 118 (–NH···O, 1.84 Å) as H-bond acceptor. The –NH groups in Gln 66 and Leu 65 are linked separately with one hydroxyl oxygen of DS38 by hydrogen bonds at a distance of 2.42 Å and 1.94 Å. While the oxygen atom of Glu 67 forms two similar H-bonds with the two hydroxyl hydrogen atoms in the compound (–OH···O, 1.63 Å) and (–OH···O, 1.51 Å) as H-bond acceptors, respectively. Additionally, another hydrogen bond is formed between the carbonyl oxygen of DS38 and side chain hydroxyl hydrogen of amino acid Ser 137 at a distance of 2.55 Å. Overall, these results indicate that the binding models obtained from MD are reasonable and may offer constructive suggestions to further validate the compound-target interactions by using our method.

### Herb feature mapping

**Figure 3:**
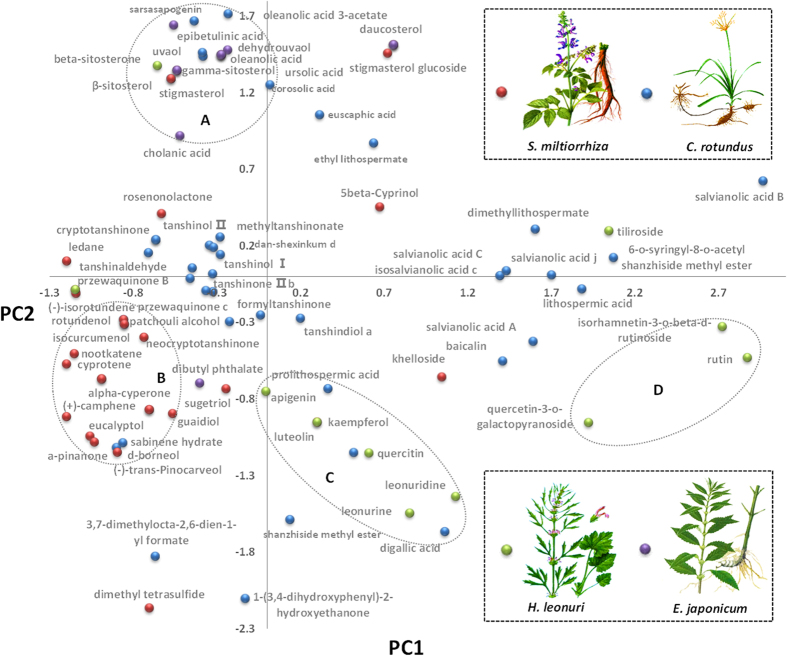
Chemical space distributions of the active components present in S. miltiorrhizae, H. leonuri, C. rotundus, and E. japonicum based on their drug-related physicochemical properties.

Generally speaking, the chemical composition of herbs will provide the building blocks of the pharmacology activities in TCM. The nature of pharmacological synergy in psychoactive herbal medicine is probably due to the bioactive compounds targeting a similar receptor or physiological system^83^. To investigate the relationship between chemical feature and pharmacology activity to some extent and explain the synergy mechanism of molecular details about why *S. miltiorrhizae* can be combined with other three herbs to administrate on different diseases, eight representative drug-related physicochemical properties including MW, nCIC, MlogP, nHDon, nHAcc, RBN, Hy and TPSA were involved, since these parameters can reflect the basic characteristics of a molecule especially its pharmacodynamic properties (Table S5)^13^. PCA was performed on the chemical structural data matrix in order to obtain an overview of the compound distribution in the molecular descriptor space and to analyze the similarities and differences between variables, as the systematic investigations of chemical space are usually used for measuring the diversity of a compound library^24^. The descriptor matrix was modeled by the first two principal components (PCs) which are significant according to cross-validation and explain a major part (87.7%) of the descriptor data variance (Table 2). The first PC (PC1) defines the combination of variables that explains the greatest amount of variation (67.7%), and each component is expected to contribute in an equal manner to PC1. The second PC (PC2) indicates the next largest amount of variation accounts for about 20.0% of the total variance. The distribution of the compounds on the first two latent variables derived by PCA is shown in Fig. 3.

As seen in Fig. 3, an overview of molecular distribution of all molecules in each herb can be visualized by the principal component analysis. According to the loading plot, the active components of *S. miltiorrhizae* are widely distributed on the whole panel, which shows that *S. miltiorrhizae* has a broad range of chemical and functional diversity. This may be consistent with the fact that *S. miltiorrhizae* is one of the most commonly used Chinese herbs in modern Chinese clinical treatment, which have anticoagulant, vasodilatory, increased blood flow, anti-inflammatory, free radical scavenging, mitochondrial protective and other activities by the pharmacological examinations^84^. It was interesting that *S. miltiorrhizae* has no evident separation with other three herbs. This phenomenon suggests that *S. miltiorrhizae* may combine with other different herbs for a diversity of ailments. For example, *S. miltiorrhizae* can cooperate with *H. leonuri*, *C. rotundus* and *E. japonicum* for treatment of CHD, dysmenorrheal and nephrotic syndrome in clinical trials, respectively.

In addition, from the scattered points, *E. japonicum*, *C. rotundus* and *H. leonuri* are relatively far from each other, which are grouped into four areas marked as groups A, B, C and D, respectively. The scattered distribution of these three different herbs shows that chemical differences may result in the differences of physicochemical activity. That also indicates that target proteins for the three herbs may be different. For instance, β-sitosterol, daucosterol, cholanic acid and oleanolic acid in *E. japonicum* (region A) have carbonic anhydrase II (CA2) as their target and exhibit effects on renal failure^85^. Progesterone receptor (PGR), which is involved in pain^86^, is hit by compound nootkatone in *C. rotundus* (region B). The androgen receptor (AR) as a target hit by the compounds leonuridine (region C) and quercetin-3-o-galactopyranoside (region D) in *H. leonuri*, functions in hypertension in male spontaneously hypertensive rats (SHR) and requires no conversion of testosterone to dihydrotestosterone^87^. To sum up, these results allow a clear discrimination of bioactive molecules in four different herbs according to the attributes that describe them. The comparing scaffolds of bioactive natural products inherent to different medicinal herbs could provide a great significance in further understanding of scaffold architectures in different herbs that might be suitable for combinatorial library design.

### Herb pairs-based pharmacological networks

**Figure 4:**
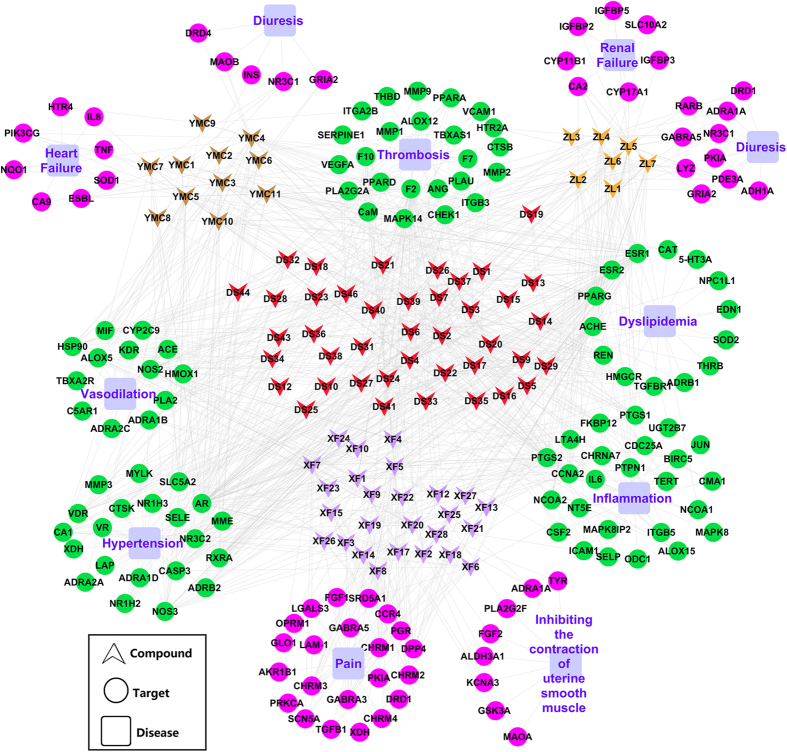
C-T-D network of three S. miltiorrhizae pairs.

It seems highly likely that TCM acts through complex mechanisms featured as multi-compound, multi-target and multi-disease. Current studies have indicated that two or more herbs interact with multiple targets simultaneously at reasonably close affinities in the biological network and then the biological system would attain new equilibrium in order to reduce the harmful impact^26^. In such an interlinked network, it would have some effects on the body through this network when a drug targets at a protein. Meanwhile, multiple drugs may connect with several proteins to affect the whole network. More importantly, it has been realized that, to be the most effective and the newest therapeutic strategies against complex diseases, drugs should target entire disease-associated network rather than single protein. Fortunately, emerging network-based approaches^13^,^15^,^16^,^17^ are becoming more and more powerful to predict and analysis such complex systems, which have always been the major objective of bioinformatics technology^88^. Networks analysis is widely applicable throughout the drug discovery and development pipeline, offering a global perspective of their physiological context without losing the key molecular details. Any type of data which linked to a gene, a compound or a protein can be recognized, visualized and analyzed by the input parsers on the networks. Particularly, the C-T-D network which links drugs, protein targets and their related diseases was used to interpret the mechanism of drug action and explore polypharmacology and predict new targets for drugs^89^. Therefore, in this section, the C-T-D network has been employed to disclose the multiple synergy actions of drugs on multiple targets that control different diseases from three *S.miltiorrhizae*-dominated herb pairs (Danshen-Yimucao, Danshen-Xiangfu, Danshen-Zelan). The detail information is as follows (Fig. 4, Table S6).

#### Danshen-Yimucao pair

The herb pair Danshen-Yimucao has been widely used to improve coronary and cerebral circulation for the therapy of cardiovascular diseases, such as CHD^90^. In this part, 44 active compounds (33 of *S. miltiorrhizae* and 11 of *H. leonuri*) and a total of 51 proteins (43 of *S. miltiorrhizae* and 31 of *H. leonuri*) as their targets are gathered to construct the C-T-D network, which links compounds, protein targets and their related diseases to interpret the mechanism of herb action for treating CHD (Fig. 4, Table S6).

*S.miltiorrhizae* identified 43 target proteins for 33 bioactive compounds. These targets are considered to have significant relationships with the pathological process of thrombosis, vasodilation, inflammation, dyslipidemia and hypertension (Table S2)^24^,^45^. For example, 12 protein targets such as thrombin (F2), coagulation factor VII (F7), coagulation factor Xa (F10) and MAPK14 are related to the thrombosis process^45^,^91^. The proteins concerned with vasodilation are angiotensin-converting enzyme (ACE), vascular endothelial growth factor receptor 2 (KDR)^92^, phospholipase A2, membrane associated (PLA2G2A) and heat shock protein HSP 90 (HSP90)^45^,^93^, the regulation of them may cause hemangiectasis, and then lower blood pressure by inhibiting the proliferation of vascular smooth muscle cells. Proteins arachidonate 15-lipoxygenase (ALOX15), Cyclin-A2 (CCNA2), prostaglandin G/H synthase 1 (PTGS1) and prostaglandin G/H synthase 2 (PTGS2) play important role in inflammatory process, control of which can prevent damage of the inflammatory factor to the blood vessel and cardiac muscle^94^,^95^.

Dyslipidemia is elevation of plasma cholesterol, triglycerides (TGs), or both, or a low high-density lipoprotein level that is identified as one of the most important modifiable risk factors for CHD^96^. Here are 8 protein targets includeing estrogen receptor beta (ESR2), renin (REN), TGF-beta receptor type-1(TGFBR1), peroxisome proliferator activated receptor gamma (PPARG), etc. to lead to the inhibition blood lipid accumulation and oxidation, prevention of circulatory disorder, increase of blood flow, and ultimately cure of dyslipidemia^97^,^98^,^99^. Nitric oxide synthase, inducible (NOS2), E-selectin (SELE), caspase-3 (CASP3), mineralocorticoid receptor (NR3C2) are all concerned with hypertension^100^,^101^.

In *H. leonuri*, a total of 31 target proteins are obtained for 11 active compounds (Table S2). Interestingly, in these targets, 23 targets are overlapped with those of *S. miltiorrhizae*, which are considered to be closely related to the pathological processes of CHD include thrombosis, dyslipidemia, vasodilation, hypertension and inflammation. For example, the serine protease F2 hit by 25 active compounds (17 of *S. miltiorrhizae*, 8 of *H. leonuri*) plays a pivotal role in the formation of obstructive blood clots, or thrombosis. The ESR2, PPARG and 3-hydroxy-3-methylglutaryl-coenzyme (HMGCR) have the potential to lower blood pressure, improve lipid profile and endothelial dysfunction, which may be responsible for dyslipidemia, while the antagonism of mineralocorticoid receptor (NR3C2) can reduce target-organ damage in hypertensive patients and improve survival in patients with cardiovascular disease^102^.

For inflammation, PTGS2 hit by 14 compounds as a key enzyme leading to the formation of prostaglandins is the target of nonsteroidal anti-inflammatory drugs^95^. Furthermore, to take target-disease-pathway information into consideration, we find that some targets are involved in some pathways. For example, PTGS1 belong to platelet aggregation inhibitor pathway, which is known to be related to inflammation and platelet aggregation^94^. Thus the compound ursolic acid in *S. miltiorrhizae* and quercitin in *H. leonuri* could act on the same target (PTGS1) in the same pathway, thereby have synergistic effect on the treatment of CHD. These results explain why Danshen-Yimucao pair has a good compatibility to combination and exhibits similar pharmacological effects on promoting blood circulation to remove blood stasis in the clinical practices.

In particular, this network also revealed that *H. leonuri* has another beneficial effects on CHD, such as heart failure and dieresis. For example, carbonic anhydrase IX (CA9) and tumor necrosis factor (TNF) are involved in the progression of heart failure, interaction with those two proteins might decrease the risk of heart failure^103^. In addition, 3 targets include glucocorticoid receptor (NR3C1), glutamate receptor 2 (GRIA2) and monoamine oxidase B (MAOB) are connected to diuresis. The compounds leonuridine, isorhamnetin-3-o-beta-d-rutinoside and beta-sitosterone binding to NR3C1 could improve renal responsiveness to atrial natriuretic peptide (ANP) by up-regulating natriuretic peptide receptor-A (NPR-A) expression in the inner medullary collecting duct (IMCD) and induce a potent diuretic action in rats with decompensated heart failure^104^.

Except for the heart-related symptoms, it is shown that edema is a common symptom for CHD patients. Diuretics are critical to the management of several commonly encountered edematous conditions, including cardiovascular disease^105^. That is the main reason why *S. miltiorrhizae* is usually combined with *H. leonuri* to treat CHD. In summary, all the results are consist with the fact that both the two herbs focus on promoting blood circulation to remove blood stasis with the weight ratio 1~1.5:1~3 *S. miltiorrhizae*: *H. leonuri*). In Danshen-Yimucao pair, the dominant one removes heat to cool blood and the complementary one alleviates water retention and detoxication, therefore the cooperation of the two herbs brings out the best in each other with the effects of activating blood circulation, stimulating meridians and alleviating water retention^90^.

#### Danshen-Xiangfu pair

Dysmenorrhea, estimated to be present in 40–50% of young women^106^, is defined as painful menstrual cramps without any evident pathology to account for them, which refers to any degree of perceived cramping pain during menstruation. In the present work, the C-T-D network that links compounds (33 of *S. miltiorrhizae*, 19 of *C. rotundus*) and 55 protein targets (43 of *S. miltiorrhizae*, 32 of *C. rotundus*) clarifies the mechanism of Danshen-Xiangfu pair in dysmenorrhea therapy (Fig. 4, Table S6).

The *S. miltiorrhizae* and *C. rotundus* share 20 common targets, which accounting for 36.4% of the total targets. As mentioned above, *S. miltiorrhizae* targets all have significant relationships with thrombosis, vasodilation, inflammation, dyslipidemia and hypertension. For instance, the carbonic anhydrase I (CA1) hit by 9 compounds is well related to the vasodilation, the control of which will lead to the improvement of endothelial and vasomotor dysfunction^107^. The overlapped targets reveal the synergy for these two herbs to eliminate blood stasis in the dysmenorrhea therapy.

Interestingly, *C. rotundus* is also found to relieve pain for binding to 7 proteins, such as PGR, Glyoxalase I (GLO1), dipeptidyl peptidase IV (DPP4) and basic fibroblast growth factor (FGF2), etc. and inhibits the contraction of uterine smooth muscle. It is suggested that spinal PGR plays an important role in neuropathic pain, and that controlling the activity of PGR may be of great importance in the treatment of neuropathic pain^86^. CAMP-dependent protein kinase inhibitor alpha (PKIA) has been reported involved in the diminished inflammation and nociceptive pain^108^. For inhibiting the contraction of uterine smooth muscle, molecules luteolin was found to target monoamine oxidase A (MAOA), which affects the secretion of the prostaglandin^109^. Moreover, evidence showed that the dehydration altered FGF2 expression patterns in arginine vasopressin-containing magnocellular neurons and neurohypophysis, supporting that the involvement of centrally-synthesized FGF2 is putatively coupled with arginine vasopressin^110^.

These findings suggest that *C. rotundus* may relieve pain, as well as regulate the synthesis of prostaglandin and vasopressin, and ultimately cures dysmenorrhea. In addition, it has been demonstrated that prostaglandin and vasopressin production in the uterine lining are up to seven times greater in women with clinically diagnosed dysmenorrhea compared with controls^111^. For these reasons, successful treatment of this disorder has been achieved with drugs that inhibit prostaglandin and vasopressin synthesis and reduce uterine hypercontractility^112^,^113^. This is also the reason why *S. miltiorrhizae* can cooperate with *C. rotundus* for the treatment of dysmenorrheal, which also explains why Danshen-Xiangfu is arranged by the weight ratio of 1~3:1 for treating dysmenorrheal disease. In a word, *S. miltiorrhizae* plays a role in dissolving blood stasis and promoting blood flow, while *C. rotundus* is responsible for inhibiting uterine contraction, thereby alleviating pain from dysmenorrhea^114^.

#### Danshen-Zelan pair

Nephrotic syndrome, a common complication of glomerular disease in children and adults, is a nonspecific kidney disease characterized by a number of signs of disorders including massive proteinuria, hypoalbuminemia, edema, and hyperlipidemia. Recently, the interests are growing in TCM as a feasible alternative therapeutic agent for the treatment of these chronic disorders and keep the body in balance to lower disease risk. For Danshen-Zelan pair^90^, a total of 39 active ingredients (33 of *S. miltiorrhizae*, 7 of *E. japonicum*) and 53 targets (43 of *S. miltiorrhizae*, 25 of *E. japonicum*) is gathered to construct the C-T-D network, which reveals the synergistic interactions of *S. miltiorrhizae* and *E. japonicum* for treating nephrotic syndrome (Fig. 4, Table S6).

Interestingly, *S. miltiorrhizae* and *E. japonicum* own 15 cooperating targets (accounting for 28.3% of the total targets), which are associated with thrombosis, vasodilation, inflammation, dyslipidemia and hypertension according to the above discussion of *S. miltiorrhizae*. For example, 10 compounds are against the CASP3 which is a member of the cysteine-aspartic acid protease (caspase) family with the function of the execution-phase of cell apoptosis. It has been reported that the activated CASP3-dependent apoptosis pathway in the rostral ventrolateral medulla (RVLM) might be involved in hypertension in stroke-prone spontaneously hypertensive rats (SHRSP)^115^. This indicates that Danshen-Zelan pair also has anticoagulant, antihyperlipidemia, antihypertensive, anti-inflammatory effects, thus affecting occurrence and progress of blood stasis symptom.

Particularly, we also find that the *E. japonicum* possesses its own specific targets, which are related to renal failure and dieresis. For renal failure, the research demonstrated that CA2 has the capacity of inducing regeneration and inhibiting apoptosis in an *in vivo* experimental model of renal ischemia-reperfusion^116^, since ischemia remains the major cause of acute renal failure in the adult population^117^. In addition, the effect of diuresis on lysozyme (LYZ) excretion should be considered in studies utilizing this enzyme as a marker of renal injury^118^.

In addition to the therapy of renal-related symptoms, diuretics are an important remedy used in patients with nephrotic syndrome for the treatment of edema. This explanation fits the fact that *S. miltiorrhizae* is often used in combination with *E. japonicum* to treat nephrotic syndrome according to the weight ratios of 1:1. This herb pair is capable of systematically controlling nephrotic syndrome through the synergistic interactions of *S. miltiorrhizae* and *E. japonicum*, as *S. miltiorrhizae* has the function of cooling blood and promoting blood circulation while *E. japonicum* can activate blood stasis and relieve edema^90^.

### Ligand-Target Analysis

**Figure 5:**
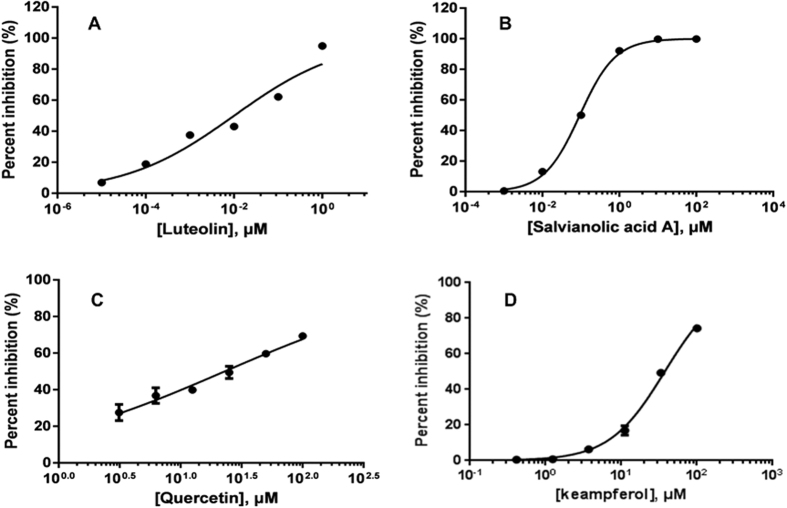
Dose-effect curves of ligands and targets.

A general idea about the specificity of an inhibitor is provided by its IC_50_ value. As shown in Table 3 and Fig. 5, the experimental IC_50_ values from the inhibition test exhibit an estimate for antagonistic effects, which are consistent with that derived from our model. Compound luteolin was tested in MAOA inhibition assays and exhibited strong activity against MAOA with IC_50_ of 0.01 μM (Fig. 5A). F10 binding study *in vitro* provided the evidence that salvianolic acid A was able to bind to the ligand and exhibit antagonistic effects (IC_50_ =  0.09 μM, Fig. 5B). Moreover, quercitin with an apparent IC_50_ of about 22.90 μM had obvious inhibitory effect on the target PIK3CG (Fig. 5C), while PTGS2 was found to have an IC_50_ of 37.39 μM for kaempferol in a characteristic manner of antagonists (Fig. 5D). These findings suggested that our method was reasonable and accurate for the evaluation of the action mode of drug-target interactions.

## Conclusions

TCM has a long history of using herbal medicine in the treatment of various diseases and is considered a complementary or alternative medical system to conventional medicine. Multi-herb recipes have frequently been used in TCM, the aim of which is to exert therapeutic efficacy collectively, including modulation pharmacological actions and/or minimization toxicity and adverse effects of the chemical ingredients of the constituent herbs. Without altering the basic therapeutic features of multi-herb formulae, herb pairs as the most fundamental form of multi-herb therapy have been frequently used to treat diseases. While the effectiveness of many herb pairs is undeniable, it is reasonable to assume that their curative effects may arise from the synergistic actions of the herbs in a specific combination. However, the molecular mechanisms of the synergistic actions of TCM herb pairs still remain unclear.

Therefore, in our study, we take three representative *S. miltiorrhizae*-dominated herb pairs (Danshen-Yimucao, Danshen-Zelan and Danshen-Xiangfu) as an example attempting to decipher the synergetic mechanism of these three pairs and further explore why different *S. miltiorrhizae* combinations have contributed to controlling various diseases. An efficient way is devoted to establishing a systematic framework based on the systems pharmacology method, which integrated *in silico* ADME evaluation, herb feature mapping, multiple targeting as well as network technology.

Our main findings are summarized as follows: 1) The combinatorial evaluation for OB, DL and HL of each herb are employed to screen the active ingredients of three *S. miltiorrhizae* pairs respectively, suggesting that chemical compositions of them have substantially different properties. 2) The identified targets related with different diseases are critical for better understanding the pharmacological mechanisms of the three *S. miltiorrhizae* -dominated pairs, as the active ingredients in each herb may target at one or multiple proteins to treat various diseases in the biological network. 3) The distribution of these active components are visualized by the herb ingredients feature mapping analysis, which is responsible for revealing the relationship of the chemistry and pharmacology of three herb pairs. 4) The generated C-T-D network clearly elucidates the molecular synergistic actions of three *S. miltiorrhizae*-dominated pairs in a holistic context and provides an in-depth explanation why different *S. miltiorrhizae* combinations can treat different diseases at the system level. 5) The *in vitro* experiment is applied to validate the reasonability of our strategy, thus to provide a credible method to investigate the complicated interaction mechanism between herbs and targets.

In conclusion, the present work has provided a systems pharmacology framework to shed light on the mystery and synergetic mechanism of different *S. miltiorrhizae* pairs. We explore the synergistic mechanism of herb formulae starting with an analysis of the simplest herb pair form, since herb pairs possess both the characteristics of complex formulae and the features of simplicity to facilitate research. The discovered mechanisms of botanic drug pairs will be not only helpful to optimize the drug combinations in multi-component and multi-targets therapeutics but also critical for developing novel drug combinations that can lead to more efficient treatments of complex diseases.

## Additional Information

**How to cite this article**: Zhou, W. *et al.* Systems pharmacology exploration of botanic drug pairs reveals the mechanism for treating different diseases. *Sci. Rep.* **6**, 36985; doi: 10.1038/srep36985 (2016).

**Publisher’s note**: Springer Nature remains neutral with regard to jurisdictional claims in published maps and institutional affiliations.

## Supplementary Material

Supplementary Information

Supplementary Table S2

Supplementary Table S3

Supplementary Table S5

Supplementary Table S6

## Figures and Tables

**Table 1:** Binding free energy estimates for each model.

Contribution	RXRA-DS38		CCNA2-YMC11		CA2-ZL7		PGR-XF7		HSP90AA1-ZL4		ESR1-DS3		NCOA2-ZL4		PLAU-XF7		MAPK14-ZL4	
	Mean	Std	Mean	Std	Mean	Std	Mean	Std	Mean	Std	Mean	Std	Mean	Std	Mean	Std	Mean	Std
Δ*E*_*ele*_	−21.27	11.57	−21.19	4.28	−45.91	5.87	−19.89	4.03	−2.89	2.92	−52.13	5.41	−9.89	3.47	−15.13	7.65	−27.04	9.41
Δ*E*_*vdw*_	−34.51	3.38	−39.85	2.80	−44.76	3.00	−43.22	2.59	−43.71	3.20	−37.40	3.70	−48.47	2.70	−38.57	2.31	−29.81	2.40
Δ*G*_*np*_	−26.93	1.08	−25.97	0.66	−36.31	0.58	−31.02	0.67	−32.79	2.04	−28.09	0.54	−28.94	0.60	−27.38	1.10	−24.88	1.14
Δ*G*_*pb*_	44.23	11.01	44.95	4.07	80.15	7.24	53.98	5.60	21.28	4.83	72.03	4.07	41.37	3.81	39.31	6.94	45.70	6.99
Δ*G*_*gas*_	−55.78	10.46	−61.04	3.38	−90.66	5.86	−63.11	4.94	−46.60	4.49	−89.53	4.15	−58.36	4.30	−53.70	7.46	−56.85	9.04
Δ*G*_*sol*_	17.30	10.82	18.98	3.98	43.85	7.15	22.97	5.65	−11.51	4.41	43.94	4.11	12.43	3.95	11.93	6.63	20.81	6.72
*−T*Δ*S*	17.26	6.79	19.12	7.04	21.81	5.51	17.04	6.39	24.94	6.36	28.02	9.14	13.12	9.76	9.82	6.89	14.55	5.96
Δ*G*_*bind*_^*a*^	−38.48	4.78	−42.05	4.34	−46.82	5.42	−40.15	3.54	−58.11	5.01	−45.60	4.23	−45.93	3.53	−41.77	4.18	−36.04	6.05
Δ*G*_*bind*_^*b*^	−21.22	—	−22.93	—	−25.01	—	−23.11	—	−33.17	—	−17.58	—	−32.81	—	−31.95	—	−21.49	—

**Table 2:** Statistical results of the PCA.

Component		Initial Eigenvalues	
	*Total*	*% of Variance*	*Cumulative %*
1	5.4	67.7	67.7
2	1.6	20.0	87.7

**Table 3:**
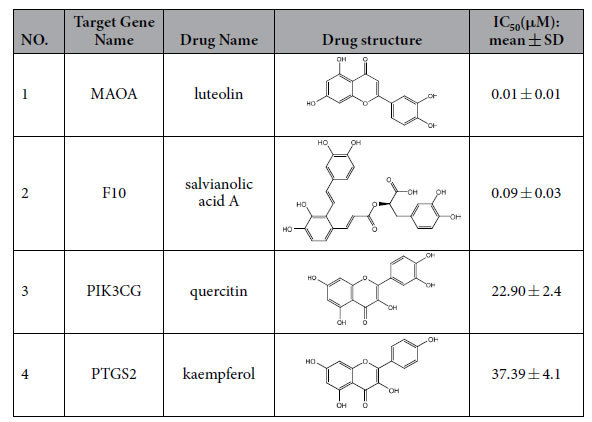
Drug structure information and IC_50_ values for the four selected compounds.
