# Supplementary material for: Systems pharmacology exploration of botanic drug pairs reveals the mechanism for treating different diseases
Source: Sci Rep. 2016 Nov 14;6:36985. doi: 10.1038/srep36985 (PMC5107896; doi:10.1038/srep36985)
Supplement: Supplementary Information [file srep36985-s1.pdf]

Systems pharmacology exploration of botanic drug pairs reveals the mechanism for treating different diseases

Wei Zhou[a,b,c,d] , Jinan Wang[a], ZiyinWu[a], Chao Huang[a] , Aiping Lu[c,d], YonghuaWang[a,b,*]

[a] Key Laboratory of Xinjiang Endemic Phytomedicine Resources, Ministry of Education; Pharmacology Department, School of Pharmacy, Shihezi University, Shihezi, Xinjiang, China.

[b] Center of Bioinformatics, Northwest A & F University, Yangling 712100, Shaanxi, China.

[c] Institute of Arthritis Research, Shanghai Academy of Chinese Medical Sciences, Guanghua Integrative Medicine Hospital / Shanghai University of T.C.M, Shanghai 200052, China.

[d] Institute of Basic Research in Clinical Medicine, China Academy of Chinese Medical Sciences, Beijing 100700, China.

*Corresponding author: YH Wang: dcpwyh@163.com. Tel. & Fax: +86-029-87092262.

Supplementary table legends

Table S1 The computational efficiency of algorithms used in our strategy.

Table S2 92 active compounds from three Danshen herb pairs and corresponding predicted OB, DL and DHL.

Table S3 All compounds, targets and related diseases information of three Danshen herb pairs.

Table S4 70 active compounds in four herbs and the corresponding calculated OB, DL and HL.

Table S5 Enriched PCA-related molecular properties terms of three Danshen herb pairs.

Table S6 The information of compound-target-disease network in three Danshen herb pairs.

Table S1 The computational efficiency of algorithms used in our strategy.

Algorithm	Software/Language	CPU Type	CPU Time	Memory
OB	Matlab	16 core 3.2G Hz	< 30 minutes	8G
DL	Matlab	16 core 3.2G Hz	< 5 minutes	8G
HL	Matlab	16 core 3.2G Hz	< 5 minutes	8G
SVM/RF	Matlab	16 core 3.2G Hz	< 4 hour	8G
Docking	Autodock	16 core 3.2G Hz	< 50 hour	8G
MD	Amber 10	16 core 3.2G Hz	～1 month	32 * 16 node

Table S4 70 active compounds in four herbs and the corresponding calculated OB, DL and HL.

NO.	Compound	Structure	OB	DL	HL	Herbs
DS1	oleanolic acid	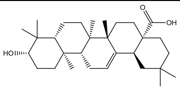	29.02	0.76	4.56	*S.miltiorrhizae*
DS2	ursolic acid	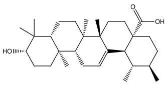	16.77	0.75	4.73	*S.miltiorrhizae*
DS3	digallic acid	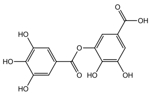	61.85	0.26	5.29	*S.miltiorrhizae*
DS4	stigmasterol	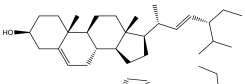	43.83	0.76	5.34	*S.miltiorrhizae*
DS5	gamma-sitosterol	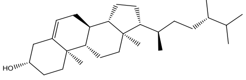	36.91	0.75	5.07	*S.miltiorrhizae*
DS11	1-(3,4-dihydroxyphenyl)-2-hydroxyethanone	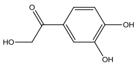	56.79	0.04	17.93	*S.miltiorrhizae*
DS12	3,7-dimethylocta-2,6-dien-1-yl formate	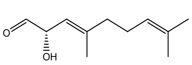	50.95	0.03	7.13	*S.miltiorrhizae*
DS14	2-(4-hydroxy-3-methoxyphenyl)-5-(3-hydroxypropyl)-7-methoxy-3-benzofurancarboxaldehyde	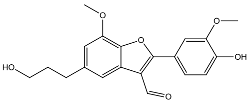	62.78	0.40	7.89	*S.miltiorrhizae*
DS15	6-o-syringyl-8-o-acetyl shanzhiside methyl ester	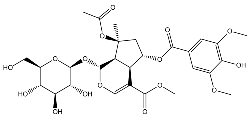	46.69	0.71	9.94	*S.miltiorrhizae*
DS16	formyltanshinone	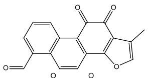	73.44	0.42	24.12	*S.miltiorrhizae*
DS17	przewaquinone B	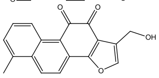	62.24	0.41	24.94	*S.miltiorrhizae*
DS19	tanshinaldehyde	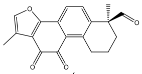	52.47	0.45	23.49	*S.miltiorrhizae*
DS20	tanshinol Ⅱ	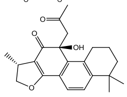	57.95	0.56	4.28	*S.miltiorrhizae*
DS21	tanshinol Ⅰ	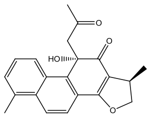	56.97	0.52	5.15	*S.miltiorrhizae*
DS22	baicalin	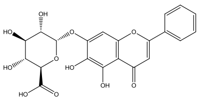	10.21	0.75	15.46	*S.miltiorrhizae*
DS23	cryptotanshinone	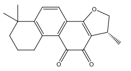	52.34	0.40	17.30	*S.miltiorrhizae*
DS24	dan-shexinkum d	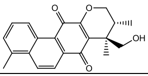	38.88	0.55	30.00	*S.miltiorrhizae*

DS25	danshen spiroketal lactone	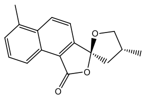	50.43	0.31	15.19	*S.miltiorrhizae*
DS26	daucosterol	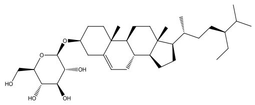	36.91	0.63	9.46	*S.miltiorrhizae*
DS28	ethyl lithospermate	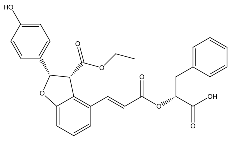	26.37	0.82	15.32	*S.miltiorrhizae*
DS29	isocryptotanshinone	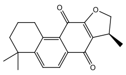	54.98	0.39	31.92	*S.miltiorrhizae*
DS30	isosalvianolic acid c	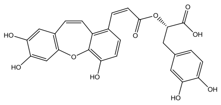	2.48	0.83	5.91	*S.miltiorrhizae*
DS31	lithospermic acid	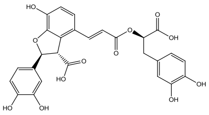	2.67	0.76	15.68	*S.miltiorrhizae*
DS32	methyltanshinonate	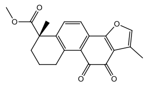	19.19	0.55	24.11	*S.miltiorrhizae*
DS33	neocryptotanshinone	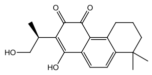	52.49	0.32	14.46	*S.miltiorrhizae*
DS34	prolithospermic acid	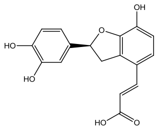	64.37	0.31	8.82	*S.miltiorrhizae*
DS35	salvianolic acid A	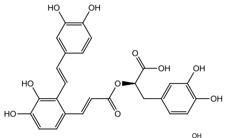	2.96	0.70	5.21	*S.miltiorrhizae*
DS36	salvianolic acid C	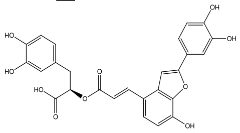	2.50	0.81	13.62	*S.miltiorrhizae*
DS37	(Z)-3-[2-[(E)-2-(3,4-dihydroxyphenyl)vinyl]-3,4-dihydroxy-phenyl]acrylic acid	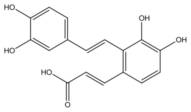	88.54	0.26	4.31	*S.miltiorrhizae*
DS38	salvianolic acid j	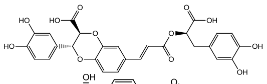	43.38	0.72	5.77	*S.miltiorrhizae*
DS39	tanshindiol a	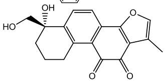	75.39	0.46	23.45	*S.miltiorrhizae*
DS40	tanshinone Ⅱb	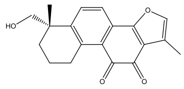	65.26	0.45	23.48	*S.miltiorrhizae*
DS41	salvianolic acid B	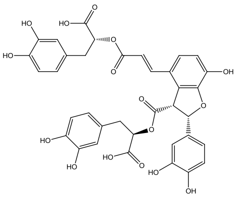	3.01	0.41	18.53	*S.miltiorrhizae*
YMC1	beta-sitosterone	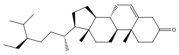	38.00	0.76	5.45	*H. leonuri*

YMC2	(−)-alloaromad endrene	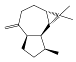	54.04	0.10	12.06	*H. leonuri*
YMC3	quercetin-3-o-galactopyranoside	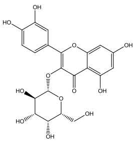	56.53	0.77	17.47	*H. leonuri*
YMC4	quercitin	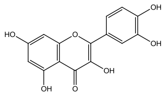	46.43	0.77	14.40	*H. leonuri*
YMC5	rutin	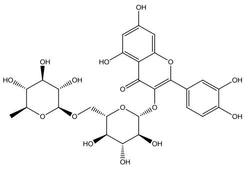	46.43	0.68	16.13	*H. leonuri*
YMC6	tiliroside	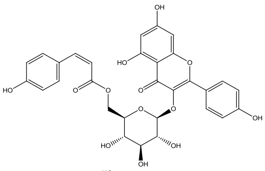	1.94	0.66	16.51	*H. leonuri*
YMC7	Isorhamnetin-3-o-beta-d-rutinoside	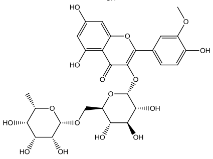	62.00	0.65	13.95	*H. leonuri*
YMC8	leonuridine	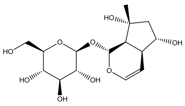	106.15	0.33	6.42	*H. leonuri*
YMC9	leonurine	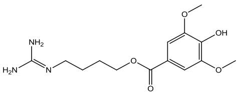	19.12	0.20	4.71	*H. leonuri*
YMC10	apigenin	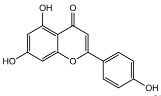	69.81	0.21	16.62	*H. leonuri*
YMC11	kaempferol	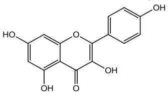	69.31	0.24	12.68	*H. leonuri*
XF1	luteolin	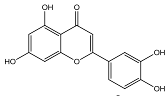	36.16	0.78	15.94	*C.rotundus*
XF4	oleanolic acid	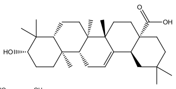	29.02	0.76	4.56	*C.rotundus*
XF5	daucosterol	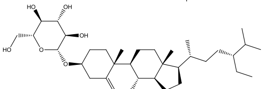	36.91	0.62	10.12	*C.rotundus*
XF6	β-sitosterol	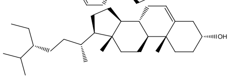	36.91	0.75	5.37	*C.rotundus*
XF7	stigmasterol	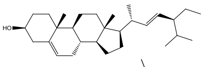	43.83	0.76	5.57	*C.rotundus*
XF9	isocurcumenol	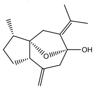	97.67	0.13	9.52	*C.rotundus*

XF11	(4aS,6R,8aS)-4a-hydroxy-4,8a-dimethyl-6-prop-1-en-2-yl-5,6,7,8-tetrahydro-1H-naphthalen-2-one	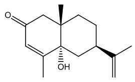	53.42	0.12	6.42	*C.rotundus*
XF12	beta-Rotunol	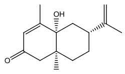	59.69	0.12	6.63	*C.rotundus*
XF14	(-)-dehydroaromadendrene	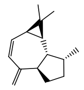	53.59	0.10	11.69	*C.rotundus*
XF15	5beta-Cyprinol	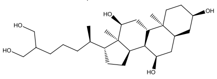	12.57	0.83	6.20	*C.rotundus*
XF16	guaidiol	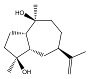	72.00	0.11	8.86	*C.rotundus*
XF18	khelloside	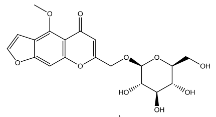	74.96	0.72	14.34	*C.rotundus*
XF19	ledane	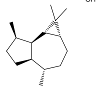	52.82	0.10	11.80	*C.rotundus*
XF20	nootkatene	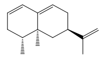	33.13	0.71	7.07	*C.rotundus*
XF21	nootkatone	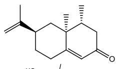	33.04	0.75	4.39	*C.rotundus*
XF23	rotundenol	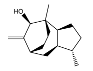	74.95	0.84	7.51	*C.rotundus*
XF24	stigmasterol glucoside	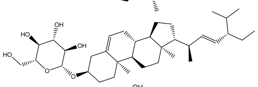	43.83	0.63	7.69	*C.rotundus*
XF25	sugetriol	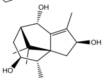	68.87	0.16	8.51	*C.rotundus*
XF26	alpha-cyperone	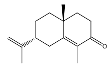	35.37	0.10	5.49	*C.rotundus*
ZL1	epibetulinic acid	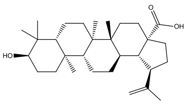	15.66	0.78	8.62	*E.japonicum*
ZL2	oleanolic acid	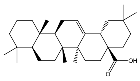	17.74	0.76	4.83	*E.japonicum*
ZL3	ursolic acid	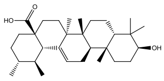	17.70	0.76	4.86	*E.japonicum*
ZL4	β-sitosterol	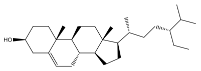	36.91	0.75	5.36	*E.japonicum*
ZL5	daucosterol	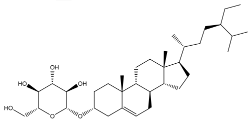	36.91	0.63	6.88	*E.japonicum*
ZL6	cholanic acid	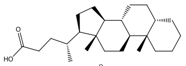	18.95	0.59	5.27	*E.japonicum*
ZL7	dibutyl phthalate	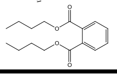	64.54	0.13	5.41	*E.japonicum*
